# Weekend Mortality in an Italian Hospital: Immediate versus Delayed Bedside Critical Care Treatment

**DOI:** 10.3390/ijerph19020767

**Published:** 2022-01-11

**Authors:** Antonio Gallo, Anna Anselmi, Francesca Locatelli, Eleonora Pedrazzoli, Roberto Petrilli, Alessandro Marcon

**Affiliations:** 1 Cardiology Department, “Magalini” Hospital, “AULSS 9 Scaligera” Veneto Region, Villafranca di Verona, 37069 Verona, Italy; 2 Cardiology Department, “Sacro Cuore Don Calabria” Hospital, Negrar, 37024 Verona, Italy; anna.anselmi77@gmail.com; 3 Unit of Epidemiology and Medical Statistics, Department of Diagnostics and Public Health, University of Verona, 37134 Verona, Italy; francesca.locatelli@univr.it (F.L.); alessandro.marcon@univr.it (A.M.); 4 Anesthesia and Intensive Care Unit, Department of Surgery, Dentistry, Pediatrics and Gynecology, University of Verona, 37134 Verona, Italy; pedrazzoli.e@libero.it; 5 Postgraduate School of Physical Medicine and Rehabilitation, Department of Neuroscience, Biomedicine and Movement Sciences, University of Verona, 37134 Verona, Italy; robi.petrilli@gmail.com

**Keywords:** critical care, consultants, weekend effect, holidays, mortality, respiratory failure

## Abstract

Background: a number of studies highlighted increased mortality associated with hospital admissions during weekends and holidays, the so–call “weekend effect”. In this retrospective study of mortality in an acute care public hospital in Italy between 2009 and 2015, we compared inpatient mortality before and after a major organizational change in 2012. The new model (Model 2) implied that the intensivist was available on call from outside the hospital during nighttime, weekends, and holidays. The previous model (Model 1) ensured the presence of the intensivist coordinating a Medical Emergency Team (MET) inside the hospital 24 h a day, 7 days a week. Methods: life status at discharge after 9298 and 8223 hospital admissions that occurred during two consecutive periods of 1185 days each (organizational Model 1 and 2), respectively, were classified into “discharged alive”, “deceased during nighttime–weekends–holidays” and “deceased during daytime-weekdays”. We estimated Relative Risk Ratios (RRR) for the associations between the organizational model and life status at discharge using multinomial logistic regression models adjusted for demographic and case-mix indicators, and timing of admission (nighttime–weekends–holidays vs. daytime-weekdays). Results: there were 802 and 840 deaths under Models 1 and 2, respectively. Total mortality was higher for hospital admissions under Model 2 compared to Model 1. Model 2 was associated with a significantly higher risk of death during nighttime–weekends–holidays (IRR: 1.38, 95% CI 1.20–1.59) compared to daytime–weekdays (RRR: 1.12, 95% CI 0.97–1.31) (*p* = 0.04). Respiratory diagnoses, in particular, acute and chronic respiratory failure (ICD 9 codes 510–519) were the leading causes of the mortality excess under Model 2. Conclusions: our data suggest that the immediate availability of an intensivist coordinating a MET 24 h, 7 days a week can result in a better prognosis of in-hospital emergencies compared to delayed consultation.

## 1. What This Paper Adds

What is already known on this subject: before the report of Cassie Aldridge et al. (Lancet 2016; 388: 178–86), no one else quantified the difference in specialist staffing between weekends and weekdays and explicitly linked the deficit in specialist staffing to the magnitude of the weekend-effect across specialties. When considering situations involving a lack of human and economic resources, policy-makers must try to identify whether an increased presence of specialists at the weekends and holidays is cost-effective.

What this study adds: this work contributes to the ongoing debate in the UK around a National Health Service providing hospital care as a 7-day service. Starting from the concept of the HiSLAC project, in our limited setting, we try to contribute to the challenging question: is the availability of hospital specialists (consultants) on holidays and weekends the central solution to the so-called weekend effect—strictly the increased mortality associated with weekend admission to hospital—or, in a broader sense, the increased mortality during weekends and holidays? We incorporated the unique aim of quantifying the role of a single consultant: the intensivist. To our knowledge, this is the first registry to quantify hospital mortality rates in emergency events when the intensivist’s involvement is in the form of a delayed consultation on weekends, holidays, or nights, versus an immediate consultation during the weekdays. Moreover, we performed a subanalysis to identify which disease is more involved in mortality. We found an excess of mortality mainly due to acute and chronic respiratory failure (ICD 9 codes 510–519).

## 2. Introduction

Since the end of the first decade of the 2000s, the public expenditure in Italy has been progressively reduced as a consequence of the economic crisis to comply with the European economic parameters imposed by the Treaties. In particular, the Maastricht Treaty (1992) was the first that bound European countries to strive for fiscal convergence to the 3% deficit to gross domestic product (GDP) ratio and the 60% debt on GDP ratio. The Stability and Growth Pact (SGP, 1997) introduced a control and sanction system and the “medium-term objective” (MTO), imposing countries with a debt to GDP ratio over 60% to cut spending. Finally, the Treaty on Stability, Coordination, and Governance in the monetary union (TSCG also known as the Fiscal Compact), introduced in 2013, provided for the inclusion of European rules in countries’ own legislative systems.

To contribute to decreased spending on health, the Italian National Health Service (SSN) sought to limit wage expenses by reducing working hours. The Fourth Sustainability Report (May 2019) of the Italian National Health Service, produced by the Italian Working Group for Evidence-Based Medicine (available on https://salviamo-ssn.it/var/uploads/contenuti/allegati/4_Rapporto_GIMBE_Sostenibilita_SSN.pdf accessed on 1 August 2021 in Italian) certified that about 25 billion euros were subtracted from the SSN between 2010 and 2015 (period of this study) due to the sum of different financial operations, and more than 12 billion euros were cut from 2015 to 2019. The increase in national health needs was +0.9% per year (equal to approximately 8·8 billion euros) during the same period, a percentage that is lower than the average annual inflation (+1.07%). In the meantime, a lack of medical specialists, due to a narrow selection of graduates admitted to the schools of specialization, has become more and more evident.

On 2 April 2012, in order to reduce spending, our general hospital shifted from an organizational model where intensivists stayed in the hospital during holidays, weekends, and at nighttime (Model 1) to a new model where intensivists were only available on call from outside the hospital (Model 2). In this way, the intensivist’s working hours were accrued only in cases of intervention and the new hires were limited.

Under Model 1, intensivists were immediately available to support a Basic Life Support and Defibrillation–trained internal medicine physician. Under Model 2, only one internist was supposed to evaluate and deal with acute critical events together with the nursing staff, and intensivists were available on call within 30 min. Such an organizational change was a unique opportunity to quantify the impact of the immediate availability of an intensive care specialist at a patient’s bedside on hospital mortality.

The aim of this study was to compare the two different emergency medicine organizational models in terms of in-hospital mortality, with a focus on the timing of admission and discharge (daytime weekdays vs. holidays, weekends, and nights), under the hypothesis that a delayed bedside critical care treatment could result in worse outcomes compared to immediate treatment in a fragile population of hospitalized patients.

## 3. Materials and Methods

### 3.1. Setting

This retrospective study was carried out in a little Italian acute care hospital (analogous to a general hospital) located in Bussolengo (Verona) with 144 inpatient beds, including departments of Emergency, Internal Medicine, Geriatric Medicine, Pulmonology, Cardiology, General Surgery, Orthopedics, Ophthalmology, Otorhinolaryngology, Urology, Obstetrics and Gynecology, Pediatrics and Neonatal Intensive Care Unit (PICU), and adult ICU. The hospital catchment area (former Azienda Ulss 22) included 37 municipalities of the province of Verona with a total of 296,330 (about 49,000 over 65) resident citizens and a population density of approximately 242 inhabitants per sq km. The Emergency Department accepted selected patients from the regional network for emergencies, while patients suffering from traumas and acute coronary syndromes were sent to a higher-level regional hospital (analogous to a district hospital). The study hospital did not have Interventional Radiology, Interventional Cardiology, Cardiac Surgery, Neurosurgery, or Dialysis facilities. During the study period (2009–2015), the annual count of Emergency Department visits ranged between 40,704 and 43,370.

**Figure 1:**
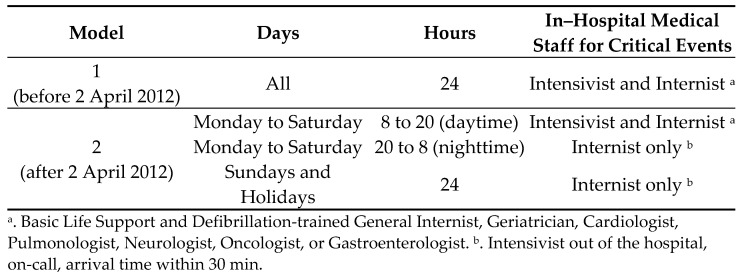
Timetable of the two models of in-hospital emergency organization.

The hospital emergency organization’s use of intensivists and internists in critical events for adult hospitalized patients before and after 2 April 2012, 8 p.m., is shown in Figure 1. During the whole study period, the pediatric specialist was the first and only medical contact for neonatal and pediatric cases, because these specialists have intensive skills and they usually manage patients autonomously in the PICU. The adult ICU was also available in the hospital throughout the study period, and it was headed by an intensivist, who dealt exclusively with patients present in the ICU ward and never moved outside to support the internist; the indication for ICU admission of a patient who experienced a critical event occurring in an ordinary ward was provided by a second intensive care consultant available (on-call) for clinical evaluation. As mentioned before, this consultation was immediate under Model 1 or delayed for up to 30 min for Model 2.

### 3.2. Data Collection and Definitions

After obtaining the local Ethics Committee’s consent, all consecutive hospital admissions from 1 January 2009 (when ICD 9 coding of discharge diagnoses was adopted in Italy) to 1 July 2015 were anonymously identified and extracted using Microsoft SQL server 2008 from the hospital discharge records database. Patients admitted to the Emergency Room but not hospitalized were not considered because these patients were always treated by the emergency physicians in both Models. Pediatric patients (under age 16 years) were excluded because they were always treated by a pediatric physician as described above.

For each admission, discharge diagnoses (classified according to the ninth revision of the International Classification of Diseases, ICD–9, clinical modification 2007), life status at discharge (alive vs. deceased), calendar day at admission/discharge, patient’s sex and age were derived. Admissions were classified into organizational Model 1 (admissions occurring during the 1185 days between 1 January 2009 and 2 April, 8 p.m, 2012) and organizational Model 2 (admissions occurring during the 1185 days after 2 April 2012, 8 p.m, until 1 July 2015). Admissions were also classified into “daytime-weekdays” admissions (from 8 a.m. to 8 p.m. during weekdays) versus “nighttime-weekends-holidays” admissions (from 8 p.m. to 8 a.m. nighttime of every day, during weekends starting from Saturday 8 p.m. to Monday 8 a.m., and during holidays 24 h from 8 a.m. to 8 a.m. next day). Primary discharge diagnoses were considered for admissions involving: neoplasms (ICD–9 codes 140–239); all diseases of the circulatory system (codes 390–459), ischemic heart disease (codes 410–414), other forms of heart disease (codes 420–429), and cerebrovascular disease (codes 430–438); all diseases of the respiratory system (codes 460–519), pneumonia and influenza (codes 480–487), and other diseases of the respiratory system (codes 510–519); diseases of the digestive system (codes 520–579); diseases of the genitourinary system (codes 580–629). Some further groups of diagnoses were not considered due to small numbers of deaths to avoid data sparseness in regression models (Supplementary Table S1).

For each hospital admission, primary and secondary discharge diagnoses were used to derive the Charlson’s comorbidity index [1].

### 3.3. Statistical Analysis

Descriptive statistics on hospital admissions were reported using means (SD) or medians (Q1–Q3) for quantitative variables and number (%) for categorical variables.

The associations between Models (2 vs. 1), the timing of admission (nighttimes and weekends vs. daytime weekdays), and mortality risk were expressed as Incidence Rate Ratios (IRR) estimated using Poisson regression models, with the life status as the outcome. The following two sets of adjustment variables: the “basic set” including patient’s sex and age in years, and season of admission, to account for the two Models covering different periods of a solar year (March–May, June–August [reference category], September–November, December–February). The “full set” also included variables that could both be interpreted as case-mix indicators at admission and outcomes at discharge: Charlson’s comorbidity index (0, 1, ≥2) and length of stay (≤7, >7 days). Since results from the two adjustment sets were quite similar, the full set was mainly considered. To account for repeated hospital admissions for the same subjects, two-level regression models were tested, with patients at level 2, and admission at level 1. However, since the variance explained by clustering was negligible, standard regression models were used for the sake of simplicity. A sensitivity analysis was also carried out defining the Model based on the date at discharge rather than the date at admission.

Finally, we assessed the association between the organizational Model and the timing of mortality using multinomial logistic regression models (estimating Relative Risk Ratios, RRR) with a categorized life status as the outcome (discharged alive, deceased during daytime weekdays, deceased during nighttimes or weekends).

The statistical analyses were performed using STATA software, release 16.1 (StataCorp, College Station, TX, USA).

Patient and Public Involvement (PPI) statement: no patient involved.

## 4. Results

During the study period, 32,900 hospital admissions of individuals aged ≥16 years were registered. Of these, 9160 programmed admissions e.g., for surgery (35 deaths) and a further 6219 admissions of people aged ≤35 years (2 deaths) were excluded to avoid statistical issues due to data sparseness. Among the remaining 17,521 admissions, 9298 (7403 patients) and 8223 (6908 patients) were registered under Model 1 and Model 2, respectively; the corresponding mean numbers of admissions per patient were 1.26 (SD 0.69) and 1.19 (SD 0.57). Under Model 2, there was a higher proportion of admissions of elderly people (≥76 years), patients with 2 or more comorbidities, and the median length of stay was longer compared to Model 1 (Table 1).

Overall, we observed 896 deaths during the nighttime–weekends–holidays, and 746 during the daytime-weekdays. The number of deaths and hospitalizations observed under organizational Model 1 and 2 for the set of discharge diagnoses considered is described in Table 2.

There was a tendency for higher mortality for patients admitted during the nighttime–weekends–holidays (IRR 1.06, 95%CI 0.95; 1.17), compared to admissions that occurred during the daytime-weekdays (Table 3).

The association became stronger and statistically significant for respiratory diseases, where we estimated some 40% increase in the number of deaths during nighttime–weekends–holidays (IRR 1.39, 95%CI: 1.16; 1.67) as well as for the subgroup labeled “other respiratory diseases” (IRR 1.41, 95%CI: 1.14; 1.73), which included acute and chronic respiratory failure (alone and associated) and other pulmonary insufficiency conditions.

Overall, there was higher mortality under Model 2 (840 deaths out of 8223 admissions) compared to Model 1 (802 deaths out of 9298 admissions) (Table 2). This translated into a 20% higher number of deaths under Model 2 compared to Model 1 in the fully adjusted analysis (IRR 1.20, 95%CI: 1.09; 1.32) (Table 4).

Similar associations were observed across all discharge diagnoses, except for digestive system diseases (IRR 0.70, 95%CI: 0.47; 1.04). The strongest associations were estimated for respiratory diseases (IRR 1.68, 95%CI: 1.42; 1.99 overall, and 1.54, 95%CI: 1.26; 1.89 for the “other respiratory diseases” subgroup), as well as for neoplasms (IRR 1.46, 95%CI: 1.16; 1.83). The sensitivity analysis defining the organizational Models based on the date of discharge was consistent with the main analysis (Table S2).

When focusing on the timing of death rather than the timing of admission, we observed that the excess risk of mortality under Model 2 (compared to Model 1) was more marked on nighttime–weekends–holidays compared to daytime-weekdays (Table 5): overall, the excess mortality was 38% during nighttime–weekends–holidays (RRR 1.38, 95%CI: 1.20; 1.59), and 12% during daytime-weekdays (RRR 1.12, 95%CI: 0.97; 1.31), and such a difference was statistically significant (*p* = 0.04). The gap in mortality risk was even more pronounced for respiratory diseases overall, as well as for the “other respiratory diseases” subgroup (Table 5).

## 5. Discussion

In this retrospective analysis of data on emergency hospital admissions in northern Italy, we found that the risk of mortality for adult in-patients was higher after a major change in the organization of emergency care occurred in 2012, from immediate availability to an intensivist permanently inside the hospital to on-call availability (arrival within 30 min) to an intensivist during the nighttimes, holidays, and weekends. We also found that mortality under the new organizational model increased significantly during nighttime weekends compared to daytime weekdays. The excess death risk was mainly related to admissions for respiratory diseases, in particular, acute and chronic respiratory failure (alone and associated) and other pulmonary insufficiency conditions.

The decreasing number of emergency medicine consultants is one of the crucial factors that have been investigated to determine hospitalized patients’ outcomes [2]. In our study, we found a higher in-patient death risk when admissions occurred during the nighttime, holidays, and weekends. Since the publication of the influential paper by Bell and Redelmeier in 2001, the “weekend effect” (whereby patients admitted to hospital over the weekend experience worse outcomes compared with patients admitted during weekdays) has been reported [3], this concept has been developed, analyzing the increased mortality rate during the weekends and holidays, irrespective of the timing of admission [4]. Many factors probably influence a worse prognosis for admissions during these critical time windows: worst clinical conditions on arrival [5,6,7,8,9,10,11]; differences in the number and expertise of hospital staff, particularly medical staff [12]; reduced access to diagnostic services, and subsequent increase of errors [13]; longer waiting time [14,15]; lower treatment appropriateness [9]; increased likelihood of hospital-acquired conditions [16]; the observation that holidays probably induced delays in seeking treatment producing a mortality increase during Christmas/New Year’s holidays [4]. The patient population assisted under Model 2 was older and presented a more severe case-mix compared to Model 1, as indicated by a higher Charlson’s comorbidity index and longer stay in hospital. Significant differences in such a broad and random population over six years were expected. While in principle these differences may be related to increased clinical complexity and mortality, our finding of higher mortality under organizational Model 2 was confirmed when adjusting for these potential confounders.

It should be noted that no other substantial organizational changes were implemented in the hospital during the observation period, in particular, in terms of the number or quality of the supply of specialists or new diagnostic tools, except for the normal turn–over of doctors, nurses, and other health care personnel.

Other studies on the weekend effect have shown that illnesses are not all involved in the same way: arrhythmia and pulmonary embolism, which were both relatively infrequent in our study, are the most affected by organizational issues [3,15,16,17]. In our study, the strongest differences between Models 1 and 2 were observed for the group of diagnoses labeled “other diseases of the respiratory system” (ICD 9 codes 510–519) which, in our population, mostly included acute respiratory failure (518.81), other pulmonary insufficiency conditions (518.82), and acute and chronic respiratory failure (518.84). These are the main critical events that mostly benefit from the early intervention of an intensivist treating the patient’s airway and giving rapid ventilatory support in order to guarantee gaseous exchange, relief from respiratory difficulty, and avoid the onset of severe acidosis, rapid multi-organ failure, and death [18,19]. The concept of recognizing signs of clinical deterioration and treating them early (the Golden Hour) was originally attributed to R.A. Cowley [20,21,22]. One of his works referred to an article by Foster, that stated the mortality rate triples for every 30 min increase from the time of injury to definitive care [23]. This 1969 article by Foster reviewed the state of helicopter transport at the time and discussed disagreement among physicians as to whether the time is an important factor in trauma care [24]. Over the years, this has become a recommendation proven by the treatment of many critical events [25,26]. For many years, in third-level hospitals, a medical emergency team (MET) has been set up, usually composed of an intensivist and a trained nurse. Data from the literature show moderate to strong evidence about the positive impact of the presence of a MET inside the hospital [23,24,25,26,27,28,29,30,31,32,33]. Observations in other Italian hospitals have highlighted better patient outcomes in organizational models equipped with a team of emergency physicians dedicated to the stabilization of emergencies [34] and better adherence to guidelines especially in a holding unit [35]. In our previous organizational model (Model 1), the intensivist, consultant to the basic life support and defibrillation (BLS–D) trained internist, was the only member of a MET–like model. In Model 2, as previously described, the intensivist dedicated to emergencies in the departments (which was different from the intensivist dedicated exclusively to the ICU) was not immediately present in the hospital, and consequently a MET–like model was missing.

The mortality excess during nighttime and holidays, as known, is also caused by short-staffing at that time and by the presence of less experienced personnel [36,37,38,39,40,41,42].

Under Model 2, we also noticed an excess of mortality for hospital admissions related to oncological diagnoses. In our study, the main diagnoses under this group were lung adenocarcinoma and primary malignant tumors with an unspecified site, followed by malignant tumors of the gastrointestinal system. It is also possible that for these conditions characterized by a poor prognosis, the immediate involvement of the intensivist could implement life-saving measures that could move the death event out of the acute care hospital setting.

The main limitation of the study, relating to the observational design, is the lack of a control group. As with all epidemiological studies, we cannot completely rule out the hypothesis that the study results are related to unmeasured patient characteristics or secular trends of another nature. Another drawback is the unavailability of a severity score for the acute event, for example, the Acute Physiology and Chronic Health Evaluation (APACHE II), to adjust the analyses for this potential confounding factor. Despite these limitations, the evidence remains strongly suggestive that delayed intervention by the intensivist can increase the risk of mortality among hospitalized patients.

## 6. Conclusions

The availability of an intensivist dedicated exclusively to hospital departments, who can treat immediately as a consultant in case of critical events 24 h, 7 days a week, can significantly reduce mortality compared to “on-call availability” from outside the hospital, especially during the nighttime, holidays, and weekends, when delayed intervention may affect patients’ prognosis. This avoidable delay affects mostly pathologies where the intensivist’s early intervention can be crucial, such as respiratory insufficiency. This scientific evidence should always be considered when attempting to save public money, especially when reorganization deals with changing a well-established, widely used organizational model.

## Figures and Tables

**Table 1:** Characteristics of the patient populations assisted under organizational Models 1 and 2 *.

Characteristic	Model 1 (before 2 April 2012)	Model 2 (after 2 April 2012)
Number of admissions	9298	8223
Age, years, mean (SD)	69.4 (16.9)	69.5 (17.3)
Age group, N (%)		
36–55 y	2066 (22.2)	1924 (23.4)
56–75 y	2821 (30.3)	2228 (27.1)
≥76 y	4411 (47.5)	4071 (49.5)
Sex, N (%)		
Female	4260 (45.8)	3719 (45.2)
Male	5038 (54.2)	4504 (54.8)
Season of admission, N (%)		
Mar–May	2341 (25.2)	2349 (28.6)
Jun–Aug	2135 (22.9)	2113 (25.7)
Sept–Nov	2175 (23.4)	1904 (23.1)
Dec–Feb	2647 (28.5)	1857 (22.6)
Timing of admission, N (%)		
daytime–weekdays	6538 (70.3)	5745 (69.9)
nighttime–weekends–holidays	2760 (29.7)	2478 (30.1)
Charlson’s comorbidity index, N (%)		
0	5709 (61.4)	5330 (64.8)
1	2117 (22.8)	1550 (18.9)
≥2	1472 (15.8)	1343 (16.3)
Length of stay, days, median (Q1–Q3)	7 (4–13)	8 (4–15)

* Statistical units for this table are hospital admissions rather than individual patients; SD, standard deviation; Q1–Q3, 1st and 3rd quartiles.

**Table 2:** Number of deaths (*n*) and hospitalizations (N) observed under organizational Models 1 and 2 for the set of discharge diagnoses considered.

Discharge Diagnosis	ICD9 Codes	Model 1, *n*/N	Model 2, *n*/N
1. All diagnoses		802/9298	840/8223
2. Neoplasms	140–239	152/550	150/377
3. Diseases of the circulatory system	390–459	190/1387	175/1134
3a. Ischemic heart disease	410–414	50/246	57/257
3b. Other forms of heart disease	420–429	114/698	100/579
3c. Cerebrovascular disease	430–438	45/263	38/177
4. Diseases of the respiratory system	460–519	259/3059	324/2077
4a. Pneumonia and influenza	480–487	59/550	44/296
4b. Other diseases of the respiratory system	510–519	181/1590	270/1424
5. Diseases of the digestive system	520–579	61/1115	40/1178
6. Diseases of the genitourinary system	580–629	18/168	22/178

**Table 3:** Incidence rate ratios (IRRs) with 95% CIs for the association between admission during nighttime–weekends–holidays (vs. daytime-weekdays) and mortality by discharge diagnosis.

Discharge Diagnosis	ICD9 Codes	Adjusted IRR (95%CI) *
1. All diagnoses		1.06 (0.95; 1.17)
2. Neoplasms	140–239	1.05 (0.80; 1.37)
3. Diseases of the circulatory system	390–459	1.09 (0.87; 1.36)
3a. Ischemic heart disease	410–414	1.26 (0.85; 1.88)
3b. Other forms of heart disease	420–429	1.10 (0.83; 1.47)
3c. Cerebrovascular disease	430–438	0.95 (0.59; 1.52)
4. Diseases of the respiratory system	460–519	1.39 (1.16; 1.67)
4a. Pneumonia and influenza	480–487	1.26 (0.82; 1.94)
4b. Other diseases of the respiratory system	510–519	1.41 (1.14; 1.73)
5. Diseases of the digestive system	520–579	1.11 (0.74; 1.66)
6. Diseases of the genitourinary system	580–629	0.78 (0.37; 1.63)

* adjusted for sex, age, season, organizational model, Charlson’s index (0, 1, ≥2), and length of stay.

**Table 4:** Incidence rate ratios (IRRs) with 95% CIs for the association between admission during organizational Model 2 (vs. Model 1) and mortality by discharge diagnosis.

Discharge Diagnosis	ICD9 Codes	IRR (95%CI) with Basic Adjustment *	IRR (95%CI) with Full Adjustment **
1. All diagnoses		1.17 (1.06; 1.29)	1.20 (1.09; 1.32)
2. Neoplasms	140–239	1.44 (1.14; 1.80)	1.46 (1.16; 1.83)
3. Diseases of the circulatory system	390–459	1.16 (0.94; 1.43)	1.18 (0.96; 1.45)
3a. Ischemic heart disease	410–414	1.00 (0.68; 1.48)	0.95 (0.65; 1.41)
3b. Other forms of heart disease	420–429	1.10 (0.84; 1.44)	1.19 (0.91; 1.57)
3c. Cerebrovascular disease	430–438	1.31 (0.84; 2.03)	1.09 (0.69; 1.71)
4. Diseases of the respiratory system	460–519	1.67 (1.41; 1.96)	1.68 (1.42; 1.99)
4a. Pneumonia and influenza	480–487	1.26 (0.85; 1.87)	1.29 (0.86; 1.94)
4b. Other diseases of the respiratory system	510–519	1.59 (1.32; 1.93)	1.54 (1.26; 1.89)
5. Diseases of the digestive system	520–579	0.61 (0.41; 0.92)	0.70 (0.47; 1.04)
6. Diseases of the genitourinary system	580–629	1.08 (0.57; 2.03)	1.20 (0.62; 2.32)

* adjusted for sex, age, season, and timing of admission (daytime-weekdays/nighttime–weekends–holidays). ** adjusted for sex, age, season, timing of admission, Charlson’s index (0, 1, ≥2), and length of stay.

**Table 5:** Relative Risk Ratios (RRRs) with 95% CIs for the association between admission during organizational Model 2 (vs. Model 1) and nighttime or daytime mortality by discharge diagnosis.

		Nighttime Mortality (vs. Discharged Alive)	Daytime Mortality (vs. Discharged Alive)	
Discharge Diagnosis	ICD9 Codes	Adjusted RRR (95%CI) *	Adjusted RRR (95%CI) *	*p* Value **
1. All diagnoses		1.38 (1.20; 1.59)	1.12 (0.97; 1.31)	0.04
2. Neoplasms	140–239	1.96 (1.38; 2.78)	1.64 (1.11; 2.43)	0.46
3. Diseases of the circulatory system	390–459	1.32 (0.98; 1.79)	1.15 (0.82; 1.62)	0.52
3a. Ischemic heart disease	410–414	1.18 (0.62; 2.23)	0.76 (0.40; 1.46)	0.28
3b. Other forms of heart disease	420–429	1.36 (0.92; 2.01)	1.14 (0.72; 1.81)	0.54
3c. Cerebrovascular disease	430–438	1.46 (0.73; 2.93)	0.94 (0.45; 1.98)	0.36
4. Diseases of the respiratory system	460–519	2.35 (1.83; 3.03)	1.62 (1.25; 2.10)	0.03
4a. Pneumonia and influenza	480–487	1.46 (0.80; 2.66)	1.44 (0.73; 2.82)	0.98
4b. Other diseases of the respiratory system	510–519	2.33 (1.71; 3.19)	1.42 (1.05; 1.93)	0.02
5. Diseases of the digestive system	520–579	0.57 (0.33; 1.00)	0.79 (0.41; 1.52)	0.44
6. Diseases of the genitourinary system	580–629	1.25 (0.44; 3.57)	1.32 (0.47; 3.70)	0.94

* adjusted for sex, age, season, the timing of admission, Charlson’s index (0, 1, ≥2), and length of stay (days). ** *p*-value obtained using Wald test for the null hypothesis that associations for nighttime and daytime mortality are not different.

## Data Availability

The data presented in this study are available on request from the corresponding author. The data are not publicly available due to confidentiality issues.

## Supplementary Materials

The following supporting information can be downloaded at: https://www.mdpi.com/article/10.3390/ijerph19020767/s1, Table S1. Diagnoses groups not considered for the analyses due to small numbers of death events. Table S2. Incidence rate ratios (IRRs) with 95%CIs for the association between admission during organizational Model 2 (vs. Model 1) and mortality, by discharge diagnosis. Sensitivity analysis with the Models coded on the basis of the date of discharge. Adjusted for sex, age, season, admission day, Charlson’s index (0, 1, ≥2), and length of stay (days).
