# Supplementary material for: Weekend Mortality in an Italian Hospital: Immediate versus Delayed Bedside Critical Care Treatment
Source: Int J Environ Res Public Health. 2022 Jan 11;19(2):767. doi: 10.3390/ijerph19020767 (PMC8776160; doi:10.3390/ijerph19020767)
Supplement: Supplementary file 1 [file ijerph-19-00767-s001.zip › ijerph-1505950-supplementary.pdf]

Supplementary material

Antonio Gallo, Anna Anselmi, Francesca Locatelli, Eleonora Pedrazzoli, Roberto Petrilli and Alessandro Marcon. Weekend Mortality in an Italian Hospital: Immediate Versus Delayed Bedside Critical Care Treatment. Int. J. Environ. Res. Public Health 2022

Table S1. Diagnoses groups not considered for the analyses due to small numbers of death events.

Discharge diagnosis	ICD9	N deaths
Infectious and parasitic diseases	1–139	13
Endocrine, nutritional and metabolic diseases, and immunity disorders	240–279	16
Diseases of the blood and blood–forming organs	280–289	12
Mental disorders	290–319	1
Diseases of the nervous system and sense organs	320–389	13
Complications of pregnancy, childbirth, and the puerperium	630–680	0
Diseases of the skin and subcutaneous tissue	680–709	2
Diseases of the musculoskeletal system and connective tissue	710–739	5
Congenital anomalies	740–759	0
Certain conditions originating in the perinatal period	760–779	0
Symptoms, signs, and ill–defined conditions	780–799	40
Injury and poisoning	800–999	0
External causes of injury and supplemental classification	V01–V83	2

Table S2. Incidence rate ratios (IRRs) with 95%CIs for the association between admission during organizational Model 2 (vs Model 1) and mortality, by discharge diagnosis. Sensitivity analysis with the Models coded on the basis of the date of discharge. Adjusted for sex, age, season, admission day, Charlson's index (0, 1, ≥2), and length of stay (days).

Discharge diagnosis	ICD9	IRR (95%CI)
1. All diagnoses		1.22 (1.10; 1.34)
2. Neoplasms	140–239	1.41 (1.12; 1.77)
3. Diseases of the circulatory system	390–459	1.22 (1.00; 1.51)
3a. Ischemic heart disease	410–414	0.97 (0.66; 1.44)
3b. Other forms of heart disease	420–429	1.25 (0.96; 1.65)
3c. Cerebrovascular disease	430–438	1.18 (0.75; 1.86)
4. Diseases of the respiratory system	460–519	1.70 (1.43; 2.02)
4a. Pneumonia and influenza	480–487	1.29 (0.86; 1.93)
4b. Other diseases of the respiratory system	510–519	1.55 (1.27; 1.91)
5. Diseases of the digestive system	520–579	0.72 (0.48; 1.08)
6. Diseases of the genitourinary system	580–629	1.16 (0.60; 2.25)
